# Experience and Preparedness of Primary Care Dentistry Residents in Dental Care Delivery to Rural Communities in South Texas

**DOI:** 10.1002/cre2.70331

**Published:** 2026-04-26

**Authors:** Rochisha Singh Marwaha, Sohini Dhar, Suman Challa

**Affiliations:** ^1^ Department of Comprehensive Dentistry, Dental Public Health Residency Program, School of Dentistry UT Health San Antonio San Antonio Texas USA; ^2^ Department of Comprehensive Dentistry School of Dentistry, UT Health San Antonio San Antonio Texas USA; ^3^ Strategic Initiatives, Dental Public Health Residency Program, Department of Comprehensive Dentistry, School of Dentistry UT Health San Antonio San Antonio Texas USA

**Keywords:** advanced education in general dentistry, health workforce training, oral health, pediatric dentistry, rural dental care

## Abstract

**Objectives:**

To assess dental residents' self‐perceived preparedness to provide dental care and their experiences delivering care in rural communities in South Texas.

**Materials and Methods:**

This cross‐sectional study evaluates advanced education in general dentistry (AEGD, *n* = 19) and pediatric dentistry (PD, *n* = 20) residents' preparation for rural dentists and experiences providing dental care in rural South Texas. Residents participated in a rural dental practice management workshop, followed by clinical rotations at rural community health centers (CHCs) in South Texas. At the conclusion of each CHC rotation, residents completed a Resident's Online Activity Report (ROAR) reflecting on their preparation for rural dental practice, ability to manage patients with complex healthcare needs, and understanding of perceived barriers to care and access issues in rural communities. Descriptive analyses were conducted to summarize residents' patient encounters, preparation to treat patients with complex healthcare needs, and evaluation of rural clinical rotation experience. Open‐ended responses were summarized narratively.

**Results:**

Primary care dentistry residents participated in 78 rural rotations and completed 74 ROARs (94.9% rate). AEGD residents self‐reported (rating system: 1 = no preparation, 4 = excellent preparation) that they were best prepared to treat dental pain (3.54) and manage patients with tobacco use (3.17), while PD residents cited being well prepared to manage early childhood caries (3.76) and dental and facial trauma (3.68). Overall, PD residents (4.19) had a higher mean rating for clinical rotations at rural CHCs than AEGD (4.01). Residents indicated that the most useful parts of the rotations were the formulation of treatment plans guided by specialist faculty, using available resources to provide dental care, and understanding challenges to receiving dental care in rural areas.

**Conclusions:**

Didactic education in rural dental practice combined with clinical rotations at rural CHCs across South Texas, improved primary care dentistry residents' understanding of challenges faced by rural populations and enhanced their clinical and patient management skills, better preparing them to care for populations with high burden of oral disease and complex healthcare needs.

## 1 Introduction

Almost one‐fifth of the population of the United States lives in rural areas, and two in five rural Americans lack access to dental care (National Organization of State Offices of Rural Health 2013). These individuals have distinct health (including oral health) challenges, since profound disparities exist in oral health and access to dental care among rural communities (Skillman et al. 2010; Doescher et al. 2009). Compared to their urban counterparts, adults living in rural communities are more likely to have untreated dental caries (31.7% vs. 25.2%), a higher percentage of periodontal disease (27.9% vs 18.7%), and almost twice as likely to be edentulous (16.3% vs. 8.8%) (Skillman et al. 2010). Additionally, among children, those residing in rural areas are more likely to have fair or poor oral health (7.3% vs. 6.6%) and less likely to receive preventive treatment such as fluoride varnish (46.6% vs. 52.5%) and dental sealants (19.5% vs. 22%) compared to those in urban communities (Skillman et al. 2010; Doescher et al. 2009).

The causes of poor oral health outcomes in rural communities are multifactorial—lack of dentists and trained specialists, longer travel distance to access dental care, and lower rates of insurance coverage and Medicaid participation among dental providers. In addition, dental anxiety, cultural norms, low health literacy, the perceived belief of the need for oral health care, and nonadherence to preventive oral health behaviors may also contribute to rural–urban disparities in oral health outcomes (Skillman et al. 2010; Doescher et al. 2009).

According to the 2021 Oral Health in America: Advances and Challenges report from the National Institute of Dental and Craniofacial Research (NIDCR), almost 60 million Americans reside in rural areas. Nearly 50 million people across the United States live in Dental Health Professional Shortage Areas (D‐HPSAs), where access to oral health care is limited—especially for low‐income individuals, Hispanic populations, children covered by CHIP and Medicaid, migrant and seasonal farmworkers, rural residents, and people with disabilities. These underserved groups often forgo basic dental care due to lack of nearby providers, inability to pay, and low oral health literacy.

In 2020, Texas had 307 D‐HPSAs across 99 counties, affecting more than 4 million people. Many of these counties are rural or federally designated frontier areas, lacking the population base to sustain dental practices and infrastructure. Texas ranked 47th nationally in the number of dentists needed (*n* = 445) to eliminate these shortages. In South Texas, 23 of the 38 counties are designated D‐HPSAs, requiring at least 100 additional dentists to meet the region's needs (Advisory Committee on Training in Primary Care Medicine and Dentistry 2020). There is only one dentist for every 2764 Texans, one of the lowest provider‐patient ratios in the nation. More than 20% of Texas counties (*n* = 59) have no dentists, another 12% (*n* = 36) have only one dentist, and over 33% have no dentists who are Medicaid providers (Texas Department of State Health Services 2019). In Texas, per capita, metropolitan counties had 2.1 times as many dentists as non‐metropolitan counties (Health Professions Resource Center 2020). Moreover, with a large proportion of Texas dentists retiring or exceeding the retirement age, this poses a powerful threat to the sustainability of the rural dental workforce in Texas (Professionally Active Dentists 2023). Overall, the number of dentists per 100,000 population in Texas trails the national average substantially, and the shortage is particularly acute in the Border Region of South Texas, a large geographic area with a population of 5 million, where the dental workforce shortage is a profound barrier to oral health care access. Dentists per 100,000 population are 60.9 nationally and 48.7 in Texas (American Dental Association, Health Policy Institute 2020). Additionally, rural populations also have higher proportions of elderly, fewer economic resources, lower insurance rates, and there is a need to identify established and innovative programs and adopt necessary policy changes to help reach these communities (Henderson‐Frost et al. 2022).

An adequate and equitably distributed force of health workers is critical for achieving an optimal healthcare system (Buchan et al. 2013; World Health Organization 2006; 2010; 2011). The World Health Organization (WHO) has issued global recommendations to improve the recruitment and retention of the healthcare workforce, particularly in rural and underserved areas (World Health Organization 2011). Of the 16 recommended interventions, successful ones in many countries have involved recruitment of students from rural backgrounds and establishment of teaching programs that encourage students to include rural experiences during their training (Buchan et al. 2013; World Health Organization 2006; 2010).

Following WHO's recommendations and to address the current and projected shortages of dental providers in rural South Texas, the Primary Care Dentistry for South Texas (PCDST) Project was launched at the University of Texas Health San Antonio School of Dentistry (UTHSA‐SoD). The goal of this project is to increase the number of primary care dentists and expand their capacity to care for patients in isolated rural communities, especially those with complex health conditions co‐occurring with oral health problems. To achieve this goal, advanced education in general dentistry (AEGD) and pediatric dentistry (PD) residents at UTHSA‐SoD receive didactic education on rural practice management and hands‐on clinical experiences at rural community health centers (CHCs) in South Texas. The objective of this study is to explore dental residents' self‐perceived preparedness and readiness to deliver dental care and services in rural South Texas, with a particular focus on the challenges encountered and insights gained during their clinical rotations in these underserved areas.

## 2 Methods

This study was classified by the Institutional Review Board at UTHSA as nonregulated research conducted for purposes of program evaluation (IRB #20210394NRR). This cross‐sectional study recruited AEGD (*n* = 19) and PD (*n* = 20) residents at UTHSA‐SoD. All residents participating in this program attended a virtual rural dental practice management workshop. The 2‐h session provides an overview of dental career pathways in rural communities, followed by an interactive session among the dental residents and faculty to discuss opportunities for rural dental practice. Additionally, the faculty guides residents in better understanding how to successfully operate a rural dental clinic and discuss steps toward practice ownership. Following the didactic session, 87 clinical rotation days were scheduled for both AEGD and PD residents at designated rural CHCs in Luling, Cotulla, Devine, and Pearsall. Nine rotations were canceled due to no patient shows or at the program's request. In total, 78 rural rotations were completed during the academic year. AEGD residents provided dental care 2 days each month at the Luling CHC, while PD residents rotated 6 days per month, spending 2 days each at Cotulla, Devine, and Pearsall CHCs.

The CHCs where the PCDR residents completed their clinical rotations deliver high‐quality healthcare to individuals of all ages, incomes, and insurance statuses. They provide medical, dental, and mental health services to the expanding population. These clinics operate in a designated Health Professional Shortage Area (HPSA) and primarily care for low‐income patients with complex health needs. The affiliated CHC clinics are training sites for UTHSA‐SOD students and residents, preparing them to provide dental care to vulnerable and underserved populations, especially in rural areas.

A Resident's Online Activity Report (ROAR) online assessment form was created to capture residents' preparation and experiences regarding dental care provision at rural CHCs in South Texas. For the ROAR assessment, residents were asked to indicate the rotation location, number of patients treated, and the type/complexity of dental procedures in each clinical day. The team developed a list of common patient conditions encountered at the CHC, including managing patients with intellectual/developmental disabilities, and residents were asked to report the specific circumstances they encountered during the rotation day. AEGD residents were also asked whether they had received mentoring by a general dentist practicing in a rural community or had prior experience visiting a rural private practice. Additionally, the residents were provided with a list of statements regarding their experience at the CHC rotation and asked to respond to the statements with a 5‐point scale: *Strongly Disagree* (1), *Disagree* (2), *Unsure* (3), *Agree* (4), and *Strongly Agree* (5) with a maximum rating of 5.00. Moreover, residents were asked three open‐ended questions addressing the most useful part of the rural CHC rotations, how the rotations changed their perception of providing dental care in rural CHCs, and ways in which the rotations could be improved in the future.

A survey link was generated via Qualtrics and sent to all residents at the conclusion of each rotation day at the rural CHCs. They could respond to the survey via cell phones, tablets, or laptops with an average completion time of 5 min. The responses were entered into Excel files and then imported into IBM SPSS statistical software version 19 (IBM SPSS, Armonk, NY, USA) for analysis. Descriptive analyses were performed to summarize information on rotation sites, patient encounters, residents' interaction with patients' condition and circumstances, and residents' preparation and evaluation of the rotation. Mean scores were calculated individually for each scale. For the open‐ended questions, responses were reviewed in full and summarized narratively to capture the context of student reflections. Rather than applying formal qualitative coding procedures, a descriptive approach was used to identify recurring ideas and perspectives across the responses.

## 3 Results

**Figure 1:**
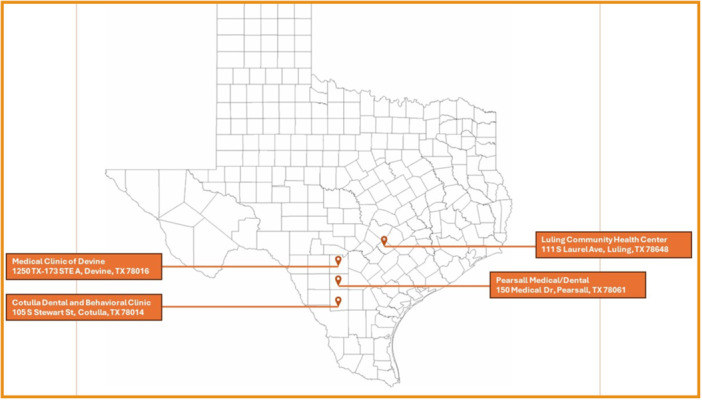
Location of community health centers (CHC) in South Texas.

Primary care dentistry residents participated in 78 rural rotations during the academic year and completed 74 ROARs (94.9% submission rate). AEGD residents submitted 24 ROARs, reported as a team of two residents, while PD residents individually submitted the remaining 50 reports. Figure 1 shows the location of the South Texas CHCs where residents completed their clinical rotations. During clinical rotations, AEGD residents saw 86 patients and PD residents saw 148 patients. The residents self‐reported that they encountered an average of 3.58 (AEGD) and 2.96 (PD) patients per rotation day.

Both AEGD and PD residents encountered unique patient circumstances and conditions during their clinical rotations at the rural CHCs. AEGD residents predominantly noted interacting with patients reporting obesity (12.8%), tobacco use (11.6%), and who were medically compromised (9.3%), while PD residents primarily treated patients with early childhood caries (15.5%), obesity (10.1%), and other circumstances (11.4%) (Table 1). Other circumstances included individuals on the autism spectrum; those whose parents held anti‐fluoride beliefs; patients with dental phobia; no prior dental visit in over 2 years; persistent pacifier use with significant overjet; low general and oral health literacy; and individuals with significant medical complications. AEGD residents reported feeling most prepared to manage dental pain (mean = 3.54), patients who use tobacco (mean = 3.17), and patients with HIV/AIDS (mean = 3.13). They felt the least prepared to manage patients with dementia (mean = 2.50), nutritional deficiencies (mean = 2.58), and cases involving abuse or neglect (mean = 2.48). PD residents indicated feeling most prepared to treat patients with early childhood caries (mean = 3.76), dental and facial trauma (mean = 3.68), and dental pain (mean = 3.50), while being least prepared to manage patients with HIV/AIDS (mean = 2.70) and those with vaping use (mean = 2.72) and opioid use (mean= 2.80) (Table 2).

**Table 1:** Primary care dentistry residents' interactions with special patient circumstances.

Residents' interaction with patient circumstances				
Circumstance/condition	PD		AEGD	
	No. of patients	% of All patients (*n* = 148)	No. of patients	% of all patients (*n* = 86)
Intellectual and Developmental Disabilities and other special needs	4	2.7%	1	1.2%
Obesity	15	10.1%	11	12.8%
Autism spectrum	3	2%	2	2.4%
Vaping use	6	4.1%	0	—
Tobacco use	4	2.7%	10	11.6%
Other substance use	0	—	5	5.8%
Early childhood caries	23	15.5%	PDR only	
Nutritional deficit/neglect	4	2.7%	4	4.6%
Dementia	AEGD only		0	—
Medically compromised	AEGD only		8	9.3%
Frail elderly	AEGD only		2	2.3%
Alcohol use	3	2.0%	5	5.8%
Dental and/or facial trauma	3	2.0%	4	4.6%
Patient abuse/neglect	0	—	3	3.5%
Orofacial pain	3	2.0%	7	8.1%
Low oral health literacy	2	1.3%	3	3.4%
Other circumstances	17	11.4%	5	5.8%
Other circumstances (breakdown)	No sedation at clinic for urgent needs (*n* = 2) Parents' anti‐fluoride (*n* = 2) Dental phobia (*n* = 2) No prior dental visit for +2 years child (*n* = 2) Pacifier habit/significant overjet (*n* = 2) Possible cysts/need imaging follow‐up (*n* = 2) Low SES; other barriers to dental care (*n* = 2) Significant medical complications (*n* = 2) Child needs comprehensive ortho (*n* = 1)		HIV/AIDS (*n* = 2) Deafness (*n* = 2) Opioid use (*n* = 1)	

**Table 2:** Evaluation of primary care dentistry residents' preparation for patient circumstances/conditions.

Preparation for patient circumstances/condition	PD (mean)	AEGD (mean)
Intellectual and Developmental Disabilities and other special needs	3.46	2.67
Obesity	3.24	2.83
HIV/AIDS	2.70	3.13
Vaping use	2.72	2.63
Tobacco use	3.02	3.17
Opioid use	2.80	2.71
ECC	3.76	N/A
Nutritional deficit	2.94	2.58
Dementia	N/A	2.50
Medically compromised		3.04
Frail elderly		2.75
Alcohol and other drug use	2.94	2.75
Dental and/or facial trauma	3.68	2.75
Pain management	3.50	3.54
Patient abuse/neglect	3.18	2.48

*Note:* Scale and point values: None (1); Minimal (2); Good (3); and Excellent (4). The maximum value is 4.00.

Residents' self‐reported preparation for rural dentistry is summarized in Table 3. AEGD residents felt more prepared to understand barriers to oral health and dental care access in rural communities (mean = 3.29), while PD residents reported greater readiness to provide dental care in these settings (mean = 3.46). Overall, residents felt confident in their ability to assess patients' oral health literacy but expressed concerns about owning or managing a dental practice in a rural area. Approximately 33% of AEGD residents reported receiving mentorship from a general dentist practicing in a rural community, while only 12.5% had previously visited a rural dental practice.

**Table 3:** Evaluation of primary care dentistry residents' preparation for rural dentistry.

Residents' ratings	PD (mean)	AEGD (mean)
Barriers to oral health in rural communities	3.38	3.29
Providing oral health care in rural communities	3.46	3.21
Owning/managing a rural dental practice	3.04	2.63
Providing care in a diverse, multi‐cultural setting	3.40	3.29
Assessing patients' oral health literacy	3.42	3.38

*Note:* Scale and point values: None (1); Minimal (2); Good (3); and Excellent (4). The maximum value is 4.00.

Resident's evaluations for clinical rural CHC rotations are shown in Table 4. Residents in both programs indicated that the rotations were well organized and supported by the clinical staff at the locations. Following the rotations, AEGD residents reported that the experience offered a new perspective on patient care compared to their current clinic settings (mean = 4.50) and enhanced their clinical and patient management skills (4.29). PD residents noted improved understanding of CHCs (4.30), greater awareness of oral health challenges in rural populations (4.28), and increased interest in rural dentistry (4.26).

**Table 4:** Evaluation of primary care dentistry residents' clinical rotations at rural CHCs.

This rotation……	PD (mean)	AEGD (mean)
was well organized and ran smoothly	4.10	3.75
was supported by helpful clinical staff	4.24	3.88
provided a different perspective on patient care than at the university clinic	4.20	4.50
increased my understanding of oral health care challenges in rural areas	4.28	4.13
expanded my understanding of community health centers	4.30	4.08
increased my understanding of dental challenges faced by patients with special needs	4.08	N/A
increased my interest in dentistry for rural populations	4.26	3.42
provided experiences with patients and oral health problems that enhanced my skills	4.04	4.29
Overall mean rating	4.19	4.01

*Note:* Scale and point values: Strongly Disagree (1); Disagree (2); Unsure (3); Agree (4); and Strongly Agree (5). The maximum rating is 5.00.

Feedback on the open‐ended questions included comments on the most useful parts of the rotations, new or different insights regarding the CHC rotation experience, and suggestions to improve future rotations (Table 5). For each prompt, themes were identified as key ideas commonly expressed as a group. Four main themes emerged as the most useful parts of the rotations: treatment planning, dental practice in rural communities, patient education, and training with specialist faculty. Residents indicated gaining experience in treating patients with complex health conditions and oral health problems while also recognizing the limitations at a rural clinic, such as minimal access to nitrous oxide for sedation. They also developed a deeper understanding of access to care challenges in rural areas and the high level of unmet dental needs. Additionally, training with dental specialists helped residents perform complex procedures and strengthen their problem‐solving skills for adequate patient management.

**Table 5:** Primary care dentistry residents open‐ended responses: 1. Most useful part of the rotation, 2. New or different insights, and 3. Improving the CHC rotations.

Prompts	Themes	Residents' responses	
		AEGD	PD
1. Most useful part of the rotation	Treatment planning	Ability to do more Endodontic procedures and third molar extractions. Getting experience in treating patients with different health and oral problems.	Treatment planning and delivery with minimal access to nitrous oxide. Able to help community in need. Doing treatment with chair‐side behavior management Understanding treatment limitations in a rural clinic; completing treatment with fewer resources.
	Dental practice in rural communities	Seeing what rural patients must do to access dental treatment. Seeing patients with many other challenges besides dental. Access to patients who need complex treatment. Getting to help patients and eliminating pain for patients in pain for so long. Providing needed care to an underserved population. Providing dental treatment to rural population.	Providing care for patients with limited access to dental providers. Learning about oral health disparities in rural areas. Devine County has no pediatric dentist, and the general dentists are not comfortable treating children. Adapting to challenging situation. Getting to visit the clinic and seeing deficiencies in dental health care among children in rural areas. There is no local pediatric dentist aside from the rural CHC. Experience with an underserved population. Importance of patient/parent education.
	Training with specialist faculty	Full‐time doctors at CHC are helpful with workflow. Treatments with limited materials; helps with problem solving. Provided experience. Ability to perform highly specialized procedures that are often not needed in urban environments.	Interacting with Pediatric Dentistry faculty. Discussion with faculty about consequences of prolonged pacifier use. Learning how to approach childhood obesity. Knowing your limitations and referring patients when needed.
2. New/different insights regarding rural dentistry	Challenges in access to care	Eye‐opener to the immense need for complex dental care in rural communities. Most patients at this clinic must drive long distances to get treatment done. It made me realize the scarcity of dental access in rural areas.	The broad range of barriers involved in access to care in small towns. Rural patients have different reasons or barriers to care for not coming in for a dental appointment. I learned more about the challenges patients from a rural environment face. There are barriers to care, which also include transportation, time, and expenses. Patients may have different reasons or barriers to care for not coming in for dental appointment. Even basic dental services are a challenge for many families at these clinics. Travel is difficult for families who do not live nearby.
	Lack of resources	There is a big need for oral health care providers with specialized training.	Everything we are used to using is not available, so we have to work with what we have. Often, rural dental practices lack appropriate equipment, so dentists must use the equipment and instruments on hand. It does not always run as efficiently as other clinics that I am used to.
	Need for adaptability/flexibility to provide dental care	Rotation allows me to learn more about dental practice in a rural community.	Adapting to clinic circumstances. You have to be more flexible. Adapting to treatment options with supplies available.
	Other challenges	There is a lot of need.	Slower paced because not everyone has easy access to dental clinic. Missed appointment rate is high. Scheduling is tough in rural clinics. Language barrier is tough to overcome.
3. Improvements to future rural/community rotations	Concerns about dental equipment and supplies	Armamentarium issues were terrible: Endo files and endo access burs are completely unorganized, lack of digital sensors caused unnecessarily long wait times. Computers, software. Increased staffing and basic materials like enough surgical hand pieces/burs. Provide more endo hooks for apex locators and sensor holders. Have more hooks for apex locators and sensor holders for RCT or the ones with a handle to hold sensor in place. Having the supplies needed to provide dental care; better equipment. Equipment was all used up even before my last appointment, and we had to use armamentarium not designed for endo to finish our last molar endo. Did not have the right equipment needed for procedures. Have more materials on hand. Provide electrical surgical handpiece.	Comments about lack of nitrous oxide: pro and con. Better organization of equipment/materials. More supplies that are appropriate for kids of different ages.
	Patient load and scheduling	Increase number of patients.	More patients. More frequent and longer rotations at community clinics. *Example: More site visits to make sure that patients know that access is available consistently*. More efficient use of time. Schedule patients closer together or have a fuller schedule.
	Effective communication	Better communication between community clinic staff could greatly increase the efficiency of the clinic. The staff had the impression that only one resident was going on rotation, and patients were scheduled only for one resident. They tried calling patients, but nobody came due to short notice.	Advertise better in community to reach more patients/parents so we could see more patients in a day. Spanish translator.

In response to the question regarding new and different insights into rural dentistry, three themes were identified: challenges in access to care, lack of resources, and the need for adaptability to provide dental care. The residents identified several challenges affecting access to care in rural settings, including transportation barriers, language differences, and treatment costs that influence patients' decisions. They noted a lack of resources in rural dental clinics and a shortage of specialized dental professionals. Furthermore, they realized the importance of adaptability and flexibility in providing dental care, given the resource limitations in rural settings. Finally, responses regarding improvements to future CHC rotations revealed three main themes: concerns related to dental equipment and supplies, patient scheduling, and effective communication. Residents suggested addressing issues related to dental and technical equipment, and supplies would be beneficial for future rotations. Additionally, improving patient scheduling process would help manage patient load more effectively and allow the residents to treat more patients during rotations. Fostering clearer communication with the clinic staff and patients would improve workflow and enhance promotion of the program, helping reach more patients in the community.

## 4 Discussion

The study explored the clinical experiences and preparedness of primary care dental residents following a rural dentistry workshop and subsequent rotations at CHCs in South Texas. The findings highlight the valuable exposure residents gained to the unique challenges of delivering oral healthcare in rural settings. These experiences not only enhanced their clinical skills but also deepened their understanding of the barriers faced by rural populations. The positive resident feedback underscores the importance of incorporating rural clinical training into dental education to better prepare future practitioners for addressing disparities in underserved communities.

Both AEGD and PD residents reported feeling well prepared to manage patients experiencing dental pain. However, AEGD residents reported gaps in knowledge and preparation when managing patients with a history of abuse/neglect, nutritional deficiencies, and dementia. In contrast, the PD residents identified challenges in treating patients with opioid abuse, vaping use, and immunocompromised individuals living with HIV/AIDS. These findings are consistent with a recent study conducted in Saudi Arabia, which revealed that dental graduates had insufficient knowledge regarding child abuse and neglect (Sulimany et al. 2021). Another cross‐sectional study examining barriers to provide clinical services for patients with substance use disorders (e.g., tobacco, alcohol, and illicit drug use) highlighted lack of knowledge and training as major challenges (McNeely et al. 2013). Previous studies among dentists also documented inadequate knowledge and perceived lack of clinical skills as barriers to treating HIV‐positive individuals (Dhanya et al. 2017; Nimbulkar et al. 2016). These data suggest the need to expand the existing curricula to better equip dental residents with the skills necessary to manage patients with complex co‐morbidities alongside dental diseases and conditions.

Following the CHC rotations, residents in both primary care dentistry programs developed a deeper understanding of the barriers to accessing oral and dental care in rural communities and the complexities involved in treating patients with multiple healthcare needs. However, they expressed limited experience and confidence in managing or owning a rural dental practice. A qualitative assessment of Canadian dental graduates similarly identified a lack of professional confidence and limited experiences in living or working in rural areas as major barriers to establishing rural dental practice (Sharifian et al. 2015). Additionally, only a limited number of our AEGD residents had previously visited a rural dentistry practice, highlighting an opportunity to expand clinical training and education in rural and remote areas where dental professionals can engage with the community and understand the unique dynamics of rural practice.

Residents responded positively to the overall learning and clinical skills development during their rural rotations. They indicated increased confidence in performing complex dental procedures and managing medically compromised patients. Importantly, the rotations broadened their understanding of oral health disparities among rural populations, enriching their clinical experience. These findings are consistent with the literature on rural clinical rotations, where dental graduates benefited by increasing their confidence and skills to serve rural populations and developing an understanding of patients’ needs (Abuzar et al. 2009; Mashabi and Mascarenhas 2011). Residents in both programs noted that adapting to the CHC environment and utilizing available resources improved their patient education skills. This finding is particularly significant given the resource limitations commonly faced by rural clinics. Moreover, the experience reinforced the importance of oral health education, preventive care, and behavior management techniques in improving patient outcomes and delivering effective dental care in rural settings. A systematic review of training program success in recruiting healthcare providers reported that programs reported that an average of 44% of providers in training pursued rural practice after graduation, with success rates ranging from 20% to 84% across 31 programs (MacQueen et al. 2018). Although these data primarily reflect physicians, similar training‐to‐practice pathways may influence rural workforce outcomes among dental providers as well.

### 4.1 Limitations

We acknowledge several limitations in the study. The findings may not be generalizable to dental residents across other advanced education dental programs nationwide. Additionally, the data collected during the initial academic year following the introduction of workshops and rotations, resident feedback over an extended period of time, may yield different results. The ROAR survey was administered only at the end of each academic year, without a baseline assessment of students' preparation prior to the workshop. This represents a limitation in the methodology and poses a threat to internal validity, as it prevents us from accurately measuring changes in student knowledge, perceptions, or skills over time. Another limitation is that the survey did not distinguish between residents' preparedness for managing complex patient conditions and performing complex clinical treatments. This distinction is important, as some complex patient conditions may require relatively simple treatment, while others involve more advanced clinical procedures.

Responses to ROAR surveys were almost twice as high among PD residents compared to AEGD residents, which could lead to a discrepancy in the mean estimates. The AEGD residents completed the ROARs in pairs, which may not adequately reflect the experiences of each resident. In the future, mandating the completion of ROAR forms by each resident will address this limitation. Additionally, scheduling clinical rotations at the CHCs emerged as another challenge due to multiple cancellations by the patients or ineffective communication between the CHC staff and program clinic coordinators, which led residents to travel to rural areas but not treat any patients due to missed appointments and last‐minute cancellations. Furthermore, fewer than 5 patients were seen each day; this may not be an effective use of a resident's time during their residency program (by contrast, they are scheduled to see 8–10 patients per day in the academic clinic). Finally, the CHCs are located 1–2 h from the main campus, so travel time needs to be accounted for when scheduling these rotations.

### 4.2 Future Steps

Our experience introducing primary care dentistry residents to rural dentistry could inform its expansion to predoctoral dental programs and other advanced dental education programs nationally. Our residents reported that addressing nutritional deficiencies, managing patients with dementia or HIV/AIDS, and providing care to patients with a history of abuse and/or neglect and substance use disorders were challenging in these rural rotations. Annual workshops for residents on these topics can be conducted, and improvements in their knowledge and skills can be evaluated.

Ultimately, the development of a primary care dentistry residency program with a rural track indicates promise as a training and educational opportunity while addressing dental needs in underserved populations who live in areas with dental professional shortages. The Harvard School of Dental Medicine has recently launched an AEGD rural track so that residents are better able to treat underserved and vulnerable populations when they join the workforce. In addition, the Regional Initiatives in Dental Education program at the University of Washington School of Dentistry trains dentists to understand and meet the oral health needs of rural and underserved populations in the region. This program builds on the existing infrastructure of the CHCs across the state and improves access to dental care in remote communities. Using these programs as models, developing similar rural track programs is the recommended next step.

## 5 Conclusion

Incorporating rural practice into an advanced education dental curriculum provides future dental specialists with didactic and clinical training to care for populations with complex healthcare needs and high oral disease burden, and improves residents' understanding of access to care challenges facing these underserved populations. Moreover, it exposes residents to opportunities to practice in nontraditional settings and increases their knowledge of and interest in pursuing a dental career in rural communities.

## Author Contributions

R.S.M. conceptualized the study, developed the methodology, contributed to data collection, and manuscript writing. S.D. assisted with data analysis and contributed to manuscript writing. S.C. assisted in the literature review, data collection procedures, and revising the manuscript. All authors reviewed and approved the final manuscript.

## Conflicts of Interest

The authors declare no conflicts of interest.

## Data Availability

The data that support the findings of this study are available on request from the corresponding author. The data are not publicly available due to privacy and ethical restrictions.
